# Draft genome sequence of an alkaliphilic drug-resistant *Burkholderia cenocepacia_DG4* strain isolated from commercial liquid detergent in Bangladesh

**DOI:** 10.1128/mra.00496-26

**Published:** 2026-07-02

**Authors:** Anika Tasnim, Nizam Uddin, Muhammad Irfanul Islam, Saiful Islam, Altaz Jashim, Farhana Yeasmin, Ria Islam

**Affiliations:** 1 Industrial Microbiology Research Division, Bangladesh Council of Scientific and Industrial Research, Chittagong, Bangladesh; 2 Biochemistry and Molecular Biology Research Division, Biomedical and Toxicological Research Institute (BTRI), Bangladesh Council of Scientific and Industrial Research, Dhaka, Bangladesh; 3 Genomic Research Laboratory, Bangladesh Council of Scientific and Industrial Research 130051, Dhaka, Bangladesh; 4 Department of Biochemistry and Biotechnology, University of Science and Technology Chittagong 185965, Chittagong, Bangladesh; 5 Department of Biochemistry and Molecular Biology, Noakhali Science and Technology University 378872, Noakhali, Bangladesh; University of Manitoba, Winnipeg, Canada

**Keywords:** soup, alkaliphilic, resistant, *Burkholderia cenocepacia_DG4*, pathogenicity

## Abstract

We presented the draft genome sequencing of *Burkholderia cenocepacia_DG4*, isolated from commercial liquid soap. The strain demonstrated tolerance up to pH 10 and exhibited phenotypic resistance to some commercial antibiotics. The genome length was 7,888,607 bp with a GC content of 67.16%, comprising 7,964 coding sequences, 55 tRNA genes, and 22 rRNA genes.

## ANNOUNCEMENT

*The Burkholderia cepacia* complex (BCC), including *Burkholderia cenocepacia*, is widely associated with soil and water (1). These organisms can influence plant health interactions and degrade both natural and xenobiotic. At the same time, BCC species are important opportunistic pathogens, especially in people with cystic fibrosis and other immunocompromised conditions. BCC species have also been documented in contaminated pharmaceutical and personal care goods (2–4).

This study involved the examination of a commercially available liquid soap during sterility testing at the BCSIR Chattogram Laboratory (22°24′49″ N, 91°49′01″ E) in 2025. The soap sample was serially diluted in 0.85% normal saline up to 10^−3^ and inoculated onto Nutrient Agar and MacConkey Agar plates. Following incubation at 37°C for 24 h, a single colony was observed on all plates. For pH tolerance assessment, the isolate was cultured in nutrient broth adjusted to different pH values, and growth was monitored after incubation at 37°C. This strain exhibited significant tolerance to alkaline environments, with growth recorded up to pH 10. This indicates a robust adaptation to detergent-rich conditions, generally deemed unfavorable for microbial viability. This isolate was also tested against a panel of 11 commercially available antibiotics and showed resistance to vancomycin, imipenem, and amoxicillin/clavulanic acid, while remaining susceptible to piperacillin/tazobactam, chloramphenicol, levofloxacin, aztreonam, trimethoprim/sulfamethoxazole, azithromycin, ceftriaxone, and co-trimoxazole.

Whole-genome sequencing (WGS) was conducted to examine its genetic characteristics, including its antibiotic resistance and virulence profiles. The culturable plate was submitted for WGS at the icddr,b Genome Center (iGC), International Center for Diarrhoeal Disease Research, Mohakhali, Dhaka-1212, Bangladesh. Genomic DNA extraction and library preparation were performed in accordance with the manufacturer’s instructions utilizing the Wizard Genomic DNA Purification Kit (Cat. A1620, Promega, Madison, WI, USA) and Illumina DNA Prep (Illumina, San Diego, CA, USA), respectively. Sequencing was performed on the Illumina NextSeq 2000 platform, producing Illumina paired-end raw reads of 300 bp. The raw reads underwent quality control utilizing FastQC v0.12 (5). Sequencing data were edited with fastp v0.23.4 (6), followed by *de novo* assembly utilizing SPAdes v3.15.5 (7, 8), and categorized using Kraken2 v2.1.3 (9). Species was finally confirmed through whole-genome sequencing (WGS) analysis, and the isolate was designated as *Burkholderia cenocepacia DG4*. The comprehensive genome analysis services provided by BV-BRC (9) resulted in a final genome of 459 contigs with a total length of 7,888,607 bp (*N*_50_: 34,760), and a GC content of 67.16% (Table 1). As ANI calculation, we have found around 98% matched with one reference strain *Burkholderia cenocepacia* strain MSMB384WGS. The isolate exhibits multidrug resistance capabilities via efflux pumps and antibiotic-inactivating enzymes: *adeF* and *amrA* facilitate the export of toxic substances from the cell, *qacG* is related to disinfectant tolerance, *OXA-1142* along with *PEN-B1* signifies beta-lactamase activity against penicillin-like antibiotics, and the *vanH* gene within a *vanF*-type cluster is linked to glycopeptide resistance. According to Pathogen Finder v2.0 (10), *Burkholderia cenocepacia_DG4* is anticipated to possess human pathogenic potential (0.9819). The presence of *B. cenocepacia* in liquid soap raises concerns about its capacity to endure extended durations within sealed commercial packaging.

**TABLE 1:** Genomic features of *Burkholderia cenocepacia_DG4* strain

Parameters	*Burkholderia cenocepacia_DG4*
Contigs	459
Genome size (bp)	7,888,607 bp
GC content (%)	67.16
Contig *L*_50_	64
Contig *N*_50_	34,760
Coverage (×)	40
Completeness (%)	92.1
CDS	7,964
tRNAs	55
rRNAs	22
Hypothetical proteins	1,861
Proteins with functional assignments	6,103
Proteins with EC number assignments	1,656
Proteins with GO assignments	1,451
Proteins with pathway assignments	1,277
Antibiotic resistance genes	11
Bacterial virulence factor	36

## Data Availability

The present whole-genome shotgun project has been deposited in DDBJ/ENA/GenBank under accession number JBXHAV000000000. The associated BioSample and BioProject identifiers are SAMN57363271 and PRJNA1455362. Raw sequencing reads are available in the Sequence Read Archive under accession number SRR38199625.
